# Gender and non-communicable diseases in Mexico: a political mapping and stakeholder analysis

**DOI:** 10.1186/s12961-024-01125-7

**Published:** 2024-04-11

**Authors:** Emanuel Orozco-Núñez, Enai Ojeda-Arroyo, Nadia Cerecer-Ortiz, Carlos M. Guerrero-López, Beatriz M. Ramírez-Pérez, Ileana Heredia-Pi, Betania Allen-Leigh, Emma Feeny, Edson Serván-Mori

**Affiliations:** 1 Center for Health Systems Research, The National Institute of Public Health, 62100 Cuernavaca, Morelos Mexico; 2 CLACSO’s Regional Working Group On International Health, Guanajuato, Leon, Mexico; 3 Center for Population Health Research, The National Institute of Public Health, Cuernavaca, Morelos Mexico; 4 The George Institute for Global Health, Sidney, Australia

**Keywords:** Gender, Non-communicable diseases, Stakeholder analysis, Political mapping, Health policy, Social protection

## Abstract

**Background:**

Mexico and other low- and middle-income countries (LMICs) present a growing burden of non-communicable diseases (NCDs), with gender-differentiated risk factors and access to prevention, diagnosis and care. However, the political agenda in LMICs as it relates to health and gender is primarily focused on sexual and reproductive health rights and preventing violence against women. This research article analyses public policies related to gender and NCDs, identifying political challenges in the current response to women’s health needs, and opportunities to promote interventions that recognize the role of gender in NCDs and NCD care in Mexico.

**Methods:**

We carried out a political mapping and stakeholder analysis during July–October of 2022, based on structured desk research and interviews with eighteen key stakeholders related to healthcare, gender and NCDs in Mexico. We used the PolicyMaker V5 software to identify obstacles and opportunities to promote interventions that recognize the role of gender in NCDs and NCD care, from the perspective of the political stakeholders interviewed.

**Results:**

We found as a political obstacle that policies and stakeholders addressing NCDs do not take a gender perspective, while policies and stakeholders addressing gender equality do not adequately consider NCDs. The gendered social and economic aspects of the NCD burden are not widely understood, and the multi-sectoral approach needed to address these aspects is lacking. Economic obstacles show that budget cuts exacerbated by the pandemic are a significant obstacle to social protection mechanisms to support those caring for people living with NCDs.

**Conclusions:**

Moving towards an effective, equity-promoting health and social protection system requires the government to adopt an intersectoral, gender-based approach to the prevention and control of NCDs and the burden of NCD care. Despite significant resource constraints, policy innovation may be possible given the willingness among some stakeholders to collaborate, particularly in the labour and legal sectors. However, care will be needed to ensure the implementation of new policies has a positive impact on both gender equity and health outcomes. Research on successful approaches in other contexts can help to identify relevant learnings for Mexico.

## Background

The intersection of gender with hegemonic social constructs and other factors such as age, race, ethnicity, socio-economic status and sexual orientation in low- and middle-income countries (LMICs) shapes the impacts of non-communicable diseases (NCDs) and perpetuates inequality in healthcare [1, 2].

In many LMICs, political discussions as they relate to health and gender primarily focus on sexual and reproductive health rights and violence against women and girls. This reflects the fiercely contested nature of women’s right to sexual and reproductive health and the persistent scale and severity of gender-based violence as an issue. However, it can also be seen as reflecting a lack of awareness of the gendered nature of NCDs and understanding of their social impacts [3–6].

Three decades ago, rates of premature death and disability resulting from diabetes, cancers and chronic kidney disease were higher for women than men. Since then, the NCD burden measured by disability-adjusted life years (DALYs) rate has become higher for men regarding diabetes, chronic cardiovascular diseases, chronic respiratory diseases and chronic kidney disease, increasing the gender gap to the detriment of men [7]. Nevertheless, the burden of disease caused by cancers and neoplasms has been consistently higher among women. The prevalence of cancers and neoplasms, chronic cardiovascular diseases and chronic kidney disease has been higher among women than among men in the last decades [7]. For men, the prevalence is higher for chronic respiratory diseases and diabetes. However, it is very important to note that the prevalence has increased steadily for both women and men. Men are more affected by alcohol and tobacco consumption and women face higher physical inactivity, all of which are major risk factors for NCDs [7].

To design and implement health policies from a gender perspective requires considering not only differential risks to health [8, 9], but the asymmetrical exercise of power, and unequal access to essential rights and social mobility opportunities [10, 11]; that is why it is necessary to review both legal and labour bases that support the labour market and the social protection systems [12]. Scarce as it is, the evidence on policies implemented to reduce gender inequalities in healthcare highlights their slow progress and minimal success. This is due, in part, to flaws in the design, implementation and financing of policies and interventions from this perspective, and also to the lack of evidence to inform specific actions, and failure to involve women in decision-making processes [13].

Policy analysis provides a way to understand how and why governments enact certain policies and their effects, focussing on the analysis of political processes and the role and involvement of key stakeholders, as well as on understanding how a change in power and the influence of these stakeholders can inform the implementation of policies, including those related to health and gender. Ultimately, this analytical approach allows us to identify the gaps in a political agenda to influence it [14]. It enables us to understand how political decisions, institutions and the biggest political factors affect the design and implementation of health policies, access to medical attention, prevention of diseases and equity in healthcare [15].

One of its recurring applications in public health refers to the complete and contextualized characterization of existing challenges and opportunities to improve the population’s health and welfare [16]. Some of the major reflections on gender and health are related to equity (recognition that each person has different circumstances and allocation of the resources and opportunities needed to reach an equal outcome) and equality (each individual or group of people has the same resources or opportunities). This is important because “inequality and disparities among gender and income (as well as ethnic) groups create a burden of psychosocial, functional and health risks, brings us to the threads of human life that create and support well-being. These threads, woven into a cloth that we call social capital, include kin and community ties and social networks” [17].

The literature on gender and health has focussed on different intersections, including political issues [18], reproductive health [19], economic impacts and forms of social and structural vulnerability [20]. However, the link between gender and NCDs has received inadequate attention, not only within healthcare systems but also in other sectors, which have not considered the social and economic impacts of NCDs or potential responses to address them [21, 22]. Gender inequality affects the access and use of health resources, and efforts to promote health to prevent NCDs can be diminished if we ignore or perpetuate gender stereotypes through low prioritization of female health within families, women’s limited access to financial resources to cover the costs and their caring responsibilities [23]. Gender-transformative health policies can become a good driver by addressing harmful gender norms, roles and relationships, while improving health [24].

Given that inequities related to NCDs—including those related to gender—manifest themselves in the form of differential health impacts, varied exposure to risk factors, social stratification and differential vulnerability, actions are required from both the health and other government ministries, as well as allies and interested actors within society (communities, private sector companies, foundations, NGOs, civil society etc.) [25]. Interventions at a structural and policy level may be required to address determinants that lie outside the health sector, but have a major impact on achieving positive health outcomes [25, 26]. Examples include improving rates of women’s employment, promoting equal pay and implementing labour policies with a gender perspective, as well as providing monetary rewards for family care work that falls mainly on women, and creating a comprehensive national care system. Evidence suggests policies could contribute very positively to reducing gender gaps related to NCDs [25, 26].

Mexico, like many other LMICs, is experiencing a growing burden of NCDs which is creating differentiated social as well as health impacts for women and men [7, 27–29]. This paper analyses public policies related to gender and NCDs in Mexico from the perspective of key stakeholders in the federal administration from 2018 to 2022. Specifically, we identified political challenges in the current response to health needs and opportunities to promote policies that strengthen attention to NCDs from a gender perspective in Mexico and other LMICs.

## Methods

We conducted a political mapping [30] of key stakeholders related to gender and NCDs in Mexico, and a stakeholder analysis [31], with data collected for the period 2018–2022. Political mapping refers to the graphic representation of the distribution of political forces related to a public problem in a certain region, country or territory. This mapping can show the distribution of power and different positions regarding a policy, which helps us to understand the political dynamics of the issue being analysed. Stakeholder analysis is a tool used to identify the people, groups, organizations or entities that may be affected by a specific action, perceived to be affected, or that could influence such an action.

We started with a structured search of key stakeholders who work with a gender perspective within the Mexican government, specialized international institutions, non-governmental organizations (NGOs) and the academic sector. This search was performed in the Google search engine, as well as on the official websites of the Secretariats of Health, Public Education, Labour, Government and Welfare, the Deputies and Senators’ Chambers, the Mexican Institute of Social Security (IMSS) and the Institute for Social Security and Services for State Workers (ISSSTE). We used the following search words: gender, policies, Mexico, health, equity, equality, medical services, medical insurance, diabetes, hypertension, cancer, gender in healthcare, health services, women, men, programmes, chronic diseases, cardiovascular diseases, and chronic respiratory diseases (see details of the search in Appendix 1).

This search allowed us to prepare a list of potential interviews. We identified 51 key stakeholders through a combination of desk research and the snowball technique [32], to whom we emailed personalized invitations, along with the interview guide and the letter of informed consent (see Appendix 2). Of those emailed, 14 people declined the interview, of whom 8 suggested other people to be interviewed. A total of 19 people did not respond to our request; with some of them we established communication, and they replied by email or telephone but stopped answering our follow-up emails or phone calls. We conducted a total of 18 interviews between August and October 2022; 17 through the Zoom platform and one person sent her answers in written form. The people interviewed (3 men and 15 women) came from the following sectors: one from an international organization advocated to women topics, seven from government institutions, three from academia, four from health-focussed NGOs (NCDs, diabetes, cancer and cardiovascular diseases), one from the private sector and two from the legislative power (parliament).

The topics addressed in the interviews were linked to the objectives of policies related to gender and NCDs, the mechanisms of inter-institutional coordination, and the allocation of resources from a gender perspective; questions are available in the interview guide (see Appendix 2). The responses of the participants were compiled, summarized and ordered by topic into tables of content, which allowed us to identify and compare all the different responses by sector. The stakeholder analysis was conducted using the PolicyMaker V5 software [33]. This tool works from templates supplied by the desk research and the information from the interviews. It works with topics such as policy content, position and power of key stakeholders, obstacles, opportunities and strategies. These last elements of the analysis came from the research team’s interpretation of the findings. Since it was not possible to interview key stakeholders from the Secretariat of Labour or the Supreme Court of Justice, the references to their positions and narratives were taken from their official websites, which are included in Table 1 [34].

**Table 1:** Institutions of the Mexican government that work with a gender perspective

Institution	URL	Contents related to gender
Mexican government	https://www.gob.mx/	- Gender equality and transversality perspective and public policies
Secretariat of Health	https://www.gob.mx/salud	- Gender equality and transversality perspective and public policies - Approach to improve reproductive rights - Cervical and breast cancer prevention - Gender violence prevention
Secretariat of Public Education	https://www.gob.mx/sep	- Gender equality and transversality perspective and public policies
		- Education model with a social inclusion perspective
Secretariat of Labour and Social Welfare	https://www.gob.mx/stps	- Gender equality and transversality perspective and public policies - Development of a “new work culture”, with sections on gender in labour relations
Secretariat of Welfare	https://www.gob.mx/bienestar	- Gender equality and transversality perspective and public policies
Chamber of Deputies	http://www3.diputados.gob.mx/	- Gender equality and transversality perspective and public policies - Legislative model with a representativity and social inclusion approach
Mexican Senate	https://www.senado.gob.mx	- Gender equality and transversality perspective and public policies
		- Legislative model with a representativity and social inclusion approach
Mexican Institute of Social Security	http://www.imss.gob.mx/	- Gender equality and transversality perspective and public policies - Coverage of NCDs and their complications, established in the labour agreement - Preventive interventions for cancer, diabetes and cardiovascular diseases
Institute for Social Security and Services for State Workers	https://www.gob.mx/issste	- Gender equality and transversality perspective and public policies
		- Coverage of NCDs and their complications, established in the labour agreement - Preventive interventions for cancer, diabetes, and cardiovascular diseases
National Institute for Women	https://www.gob.mx/inmujeres	- Gender equality and transversality perspective and public policies
		- Approach to improve reproductive rights - Cervical and breast cancer prevention - Gender violence prevention
National Centre of Gender Equality and Reproductive Health	https://www.gob.mx/salud/cnegsr	- Gender equality and transversality perspective and public policies - Approach to improve reproductive rights - Cervical and breast cancer prevention - Gender violence prevention
Supreme Court of Justice	https://www.scjn.gob.mx/	- Gender equality and transversality perspective and public policies - Application of the human rights approach to the constitutional framework

Source: Own preparation

The participation of the people interviewed was voluntary, with previous informed consent. The protocol used as a guide for this study was approved by the Research, Ethics and Biosecurity Committees of the National Institute of Public Health (ID: CI-507-2022/CB22-173).

## Results

### Government institutions that work with a gender perspective

We identified 11 institutions of the Mexican government that had in place programs and actions that applied a gender perspective (Table 1). All websites are linked to the government’s main website, and only those related to health and social security had specific actions that combine gender and health topics.

The government’s approach to addressing gender inequality highlights economic, political and labour issues, but does not mention the role of NCDs as a source of gender inequality and inequity. The National Centre of Gender Equality and Reproductive Health stood out as the institution from the Secretariat of Health that focused on the preparation of gender-driven policies to address issues such as cancer in women, gender violence and the improvement of indicators for contraception and maternal and child health for the population without social security. However, this institution does not explicitly address other NCDs.

Another governmental institution focused on health and gender issues was the National Institute for Women, which oversees monitoring of equality indicators related to health, education, labour and justice. This institution is one of the few considering either NCDs or their related impacts, such as the heavy burden of caring for elderly or ill relatives as a source of inequality for women and girls. The National Institute for Women is primarily focussed on monitoring and taking coordinated actions to prevent violence against women, and on identifying opportunities to attain greater gender equality in the labour force and political sector.

Institutions from other sectors that consider gender as a source of inequality, such as the Secretariat of Labour and Social Welfare and the National Supreme Court of Justice, did not consider NCDs as a source of inequality. We also noted the lack of assessment mechanisms across the government to determine progress in raising awareness of gender inequalities, including the absence of a gender budgeting perspective, “which entails looking at gender issues comprehensively within the budget” with the aim of addressing gender inequities [35, 36].

### Content of policies related to health and gender

The desk research on gender in health policies identified the presence of gender in health policies related to social and reproductive, and, to a lesser extent, NCD topics (Table 2). Gender equality has been defined as a central component in government policies such as the National Development Plan, the Health Sectoral Program [37, 38], and the Institutional Programs of both the Mexican Institute of Social Security [39] and the Institute for Social Security and Services for State Workers [40]. We identified the Secretariat of Welfare as one of the government institutions responsible for monitoring gender equality indicators.

**Table 2:** Content of policies on gender and health in Mexico, 2023

Objective	Priority	Mechanisms	Indicators
Promote equality between women and men through the transversal application of the Gender Perspective in all the programs projects, and actions of the government, so that it becomes part of the regular duties of the institutions of the Federal Public Administration	Low	(1) Prepare proposals to incorporate the Gender Perspective in all the plans, programmes and actions of the federal government (2) Implement methodological tools, mechanisms and procedures that allow monitoring and assessing the actions related to gender (3) Consolidate the information regarding the progress of the actions taken	(1) Actions for women’s economic autonomy (2) Actions for the reallocation of household work and care (3) Actions to access medical services and programmes (4) Action to prevent violence (5) Actions to participate in the decision-making process (6) Actions to have safe environments

Source: Own preparation using the PolicyMaker V5 software [33, 34]

Nevertheless, when we look into specific programmes, such as the one from the extinct Health Institute for Welfare (INSABI), the approach described only mentions the aim of incorporating a gender perspective in the development of projects or technical proposals [41]. Among the regulations enforced and institutions appointed to address issues of gender inequality, the most prominent issues are violence against women, ongoing since the 1970s, and the promotion and defence of sexual and reproductive rights, which started after 1980. We did not identify any similar political support or regulatory instruments aimed at addressing the impacts of NCDs.

Given the lack of explicit consideration in government policies, we noted that NCDs as a factor in gender inequality is a low-priority topic. Nor did we identify accountability mechanisms to ensure the incorporation of a gender perspective in government projects, instruments and monitoring processes more broadly. Some planning instruments, such as the General Law for Equality between Women and Men [42] and the National Policy for Equality between Women and Men, which includes the National Program for Equality between Women and Men [43], propose the monitoring of affirmative actions related to indicators of different forms of autonomy, rights compliance and the promotion of changes that modify gender inequalities in social and political sectors. However, they do not include specific actions related to NCDs.

### Key stakeholders in the gender, health and NCD sectors

Table 3 shows key stakeholders along with their position and power in relation to gender and NCDs. According to the informants from international organizations and NGOs, the development of health policies with a gender perspective was inspired by the feminist movement and various global summits on population, health, human rights and health-determining factors that were held in Rio de Janeiro (1992), Cairo (1994) and Beijing (1995). The alignment of civil activism with global and regional agendas has been essential, according to interviewees, in focusing attention on power asymmetries and the need for political action to address violence against women. An informant from an international organization said:

*“[…] One of the most important conventions, the Convention on the Elimination of All Forms of Discrimination Against Women (CEDAW, 1979), that Mexico signed, has been emblematic because it became our international regulatory framework. CEDAW has a committee with the ability to issue recommendations on the articles set forth […]” (Interview 6, Woman, international organism)*

**Table 3:** Position and power of stakeholders on gender and NCDs in Mexico, 2023

Sector	Name	Level	Support	Power
Government	Academic 1 centre	National	High	Low
	National Institute for Women (INMUJERES)	National	High	Low
	National Centre of Gender Equality and Reproductive Health (CNEGSR)	National	High	Medium
	Mexican Institute of Social Security (IMSS)	National	Low	High
	National Centre of Preventive Programs and Disease Control (CENAPRECE)	National	High	Medium
	Advisor 1	Local	High	Low
Labour	Secretary of Labour and Social Welfare (STPS)	National	Low	High
Judicial	National Supreme Court of Justice (SCJN)	National	Low	High
Legislative	Representative 1	National	Medium	High
	Representative 2	National	Medium	High
Non-governmental organizations (NGOs)	NGO 1 health and NCDs	National	High	Low
	NGO 2 diabetes	Local	Medium	Low
	NGO 3 cancer	Local	High	Low
	NGO 4 cardiovascular diseases	Local	High	Low
International Organism Academic sector	International Organism	Regional	High	Medium
	Academic 2 north	Local	High	Low
	Academic 3 south	Local	High	Low
	Advisor 2	National	High	Low
Private sector	Private sector	National	Medium	Medium

Source: Own preparation using the PolicyMaker V5 software [33, 34]

Greatest support for the proposal to formulate public policies on gender and NCDs came from representatives from the academic sector, the government, and NGOs working locally to prevent and treat cardiovascular diseases and cancer in women. Some interviewees from the government and legislative sectors highlighted the budget allocated for equality between women and men as an important instrument for progress. In this regard, one informant from the government stated the following:

*[…] Expenditures for Equality between Women and Men [of the Federal Expenditure Budget], we have the means to strengthen all the programs in terms of the inclusion of a gender perspective. The window of opportunity that is still pending is the assessment […] (Interview 12, Woman, government)*

Interviewees from the academic sector, the government and NGOs agreed that NCDs impact not only people’s health but also their financial situation because of out-of-pocket health expenditures, especially people without social security. They expressed concern for people who have had to suspend their treatment due to the cancellation (since 2019) of special funds to finance NCD treatment for those with no social security, such as the Protection Fund against Catastrophic Expenses. In this regard, an interviewee from the private sector stated the following:

*[…] It’s a pretense that most of Annex 13 [Expenditures for Equality between Women and Men] is justified with this programme [pension for the welfare of the elderly]; there are many actions that have been left undone […] There has been a setback with the disappearance of the fund for catastrophic expenses of the Popular Insurance; this fund is now used for other issues unrelated to health. The budget and the importance of a gender-focussed health service are very delicate topics […]. (Interview 16, Man, private sector)*

Significant expenses for people with no social security and the care burden for women with family members living with NCDs were highlighted as the most significant social impacts of NCDs, as mentioned in the following testimonies:

*[…] NCDs establish a huge burden of care for the families, especially for women, which prevent them from having paid work, participating in public life and developing their economic autonomy […] (Interview 12, Woman, government)*

*[…] During this 6-year term, we have heard the discourse that women are the best caretakers and that they have to stay at home taking care of their families, which implies ignoring or not wanting to see that women also get sick from NCDs […] (Interview 11, Woman, Academia)*

Interviewees from NGOs stated that they feel distrust of the government, which diminishes their ability to run their programmes. There have been several press declarations in which governmental agents expressed that “*there is no need of support coming from civil society*”, as shown in the following testimony:

*[…] We heard in the official discourse that we, the NGOs, are the enemy… Why are we seen that way? […]. (Interview 10, Man, non-profit organization)*

These perspectives contrast with a relative lack of support for policies addressing gender and NCDs among interviewees from the legislative, private, labour and judicial sectors, whose narrative and publications suggest the healthcare sector is the only one responsible for addressing NCDs and their impacts. Legislative representatives highlighted the relevance of social security through federal labour status and healthcare mechanisms, pointing out that each public insurance carrier has its own resources and services to address health and NCDs issues. Interviewees from the legislative, private, labour and judicial sectors suggested the differences in access mechanisms and medical service coverage are not relevant, stating that the government has several programmes in place to ensure access to NCD services for people with and without social security.

Regarding the health and social impacts of NCDs, interviewees from the government, academic sector, international organizations and NGOs expressed the need to raise awareness of the consequences of NCDs in terms of the care burden, lack of access to high-cost medical services and the empowerment of women to make economic and employment decisions, which do not feature in the current public policies (Table 4).

**Table 4:**
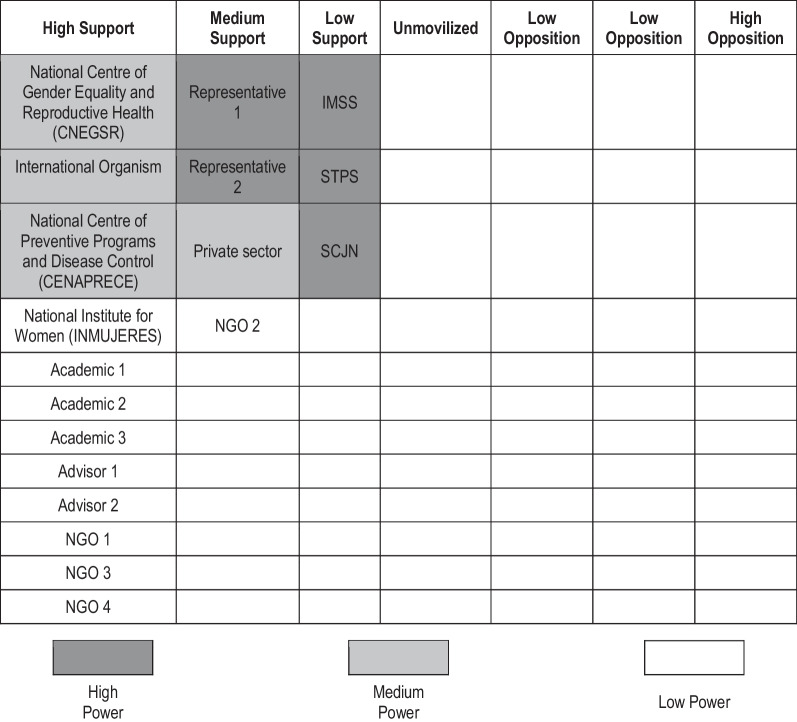
Map of stakeholders and support to NCD and gender policies in Mexico, 2022

**Source**: Own preparation using the PolicyMaker V5 software [33, 34]. IMSS, Mexican Institute of Social Security; SCJN, National Supreme Court of Justice; NGO, Non-governmental organization

These informants noted how difficult it is in the current context to develop institutional responses to the social and economic impacts highlighted above, particularly for people with no social security. In the specific cases of cervical and breast cancer, they stated that it has taken almost 20 years to develop a partnership between the Secretariat of Health, NGOs and the private sector to implement interventions aimed at improving prevention, diagnosis and treatment, especially in vulnerable populations with no ability to pay. This collaborative work was interrupted in 2018; the government’s financing for NGOs was suspended and public expenditure for the healthcare sector was reduced as part of the austerity policies of the federal government, which were aggravated by the COVID-19 pandemic.

Interviewees from the government, NGOs and the academic sector emphasized the gender-differentiated impacts of NCDs on medical expenses and care. Highlighting the role of gender in the context of care for sick people and children, which falls on women or families, these informants acknowledged the need to create a national care system that supports the unpaid work of carers, who are usually women. In this regard, an informant from an international organization said:

*[…] The Mexican government is talking about constitutional reform and secondary reforms to integrate a national care system, which is very important because it shows that there is indeed an interest from the State to create interaction among different sectors, such as education, health or labour, to complement these actions. It remains to be seen how we can pass from good political will to an actual national system […] (Interview 6, Woman, International Organism).*

The informants that saw the biggest obstacles to influencing public policies to address the gendered impacts of NCDs were part of the academic sector, the government, and NGOs, with low and medium positions in terms of political power. Informants from the private, social security, labour and justice sectors with more power expressed less support for the changes, deeming them unnecessary. The most significant difference in opinions on possible solutions for the economic and care issues came when an inter-sectoral approach was suggested; interviewees from the labour and judicial sectors, for example, saw healthcare and its impacts as the responsibility of the health sector, not their own.

When delving into possible labour and legal implications of gender and NCDs, the informants from the government, private and legislative sectors pointed out that the Mexican government’s response follows what is established in the Federal Constitution and labour legislation, which defines the type of health services that should be provided according to people’s employment and social situation. Although it was acknowledged that NCDs have effects that can be expensive and incapacitating for the people who suffer them, these interviewees did not consider it a priority to promote legal and labour changes that favour better opportunities for the people affected, or for those who care for them. In this regard, an informant from the legislative power stated the following:

*[…] It’s necessary to drive healthcare mechanisms for all the people who have a job and suffer from an NCD. We must move towards a planning process that includes not only the activities that can be scheduled, like medical appointments and examinations, but also an attention protocol or model for unexpected health events without representing losses either for the workplaces or for the patients […] (Interview 18, Woman, legislative power)*

### Obstacles and opportunities for NCD and gender policies

The obstacles identified by informants from the academic sector and NGOs for creating a bigger impact on the NCD, gender and health agendas are related to economic issues, such as budget cutbacks and limited resources due to the COVID-19 pandemic (Table 5). For example, interviewees highlighted the cancellation of public funding for NGOs working to address cancer and diabetes, and that programme expenditures from the government budget to ensure gender equality are not used to reduce inequality between men and women, nor the gender-differentiated negative consequences of NCDs [36].

**Table 5:** Obstacles and opportunities for NCD and gender policies

Sector	Obstacles	Opportunities
Academic	- Limited resources for NCD research - Limited use of research results for policies - Limited application of gender perspective in research - Lack of continuity in health and NCD programmes	- Collaborative work with the government, NGOs, legislative power and private sector - Quality and relevance of the research agenda on health and NCDs - Development of interventions to improve diagnosis and treatment - Great technical ability and skills
Government	- Absence of rights for people with no insurance - Organizational adjustments due to the pandemic and tax policies - Lack of political commitment to include a gender perspective in policies	- More inter-sectoral and collaborative work - Availability to improve diagnosis and treatment interventions - Integration of funds and areas to improve the system’s response for people with no social security - Assessment of programmes and their budgets
Judicial	- Absence of rights for people with no insurance - Perception of NCDs as only a medical problem - Limited application of gender perspective in law formulations	- Bigger involvement of female secretaries and magistrates - Political incidence in topics of public interest such as gender violence - Open to discussion on topics of the gender equality agenda - Creation of gender units
Labour	- Absence of rights for people with no insurance - Perception of NCDs as only a medical problem - Limited application of gender perspective in labour	- Open to discussion on topics of gender and equality - Promotion of actions in matters of equality and good labour practices
Legislative	- Absence of rights for people with no insurance - Limited effects of sanitary reforms on the coverage of medical services - Limited application of gender perspective in the legislative power	- Bigger involvement of female lawmakers and political representatives despite facing obstacles to their political participation - Political incidence in topics of public interest such as gender violence and the intention to push for the creation of a national care system
NGOs	- Breakdown of collaborative work with the federal government - Disappearance of special funds for financing - Lack of continuity in health and NCD programs	- Development of leadership and response capacity - Political incidence in topics of public interest such as gender violence - More support and opening from the private sector - Great technical ability and skills - Sensitization of lawmakers regarding gender equality
International Organisms	- Agenda limited to violence and sexual and reproductive health	- Signing of agreements and instruments to achieve greater gender equality - Incidence ability and collaborative work with key institutions - Great technical ability and skills
Private	- Absence of rights for people with no insurance - Limited application of gender perspective in labour - Budget cutbacks and austerity programme from the government	- Open to discussion on topics of gender and equality - More participation and leadership of women in the decision-making process in business
Social Security	- Absence of rights for people with no insurance - Limited application of gender perspective in labour	- More inter-sectoral and collaborative work

Source: Own preparation from field interviews and desk research

Interviewees from the government, judicial, labour, legislative, private and social security sectors also identified obstacles related to the lack of rights of people living with NCDs who have no social security. All the informants acknowledged that Mexico’s biggest vulnerability lies in the lack of proper and prompt access to medical services. This obstacle was combined with the perception from the judicial, labour, legislative and private sectors that healthcare issues must be seen from a medical perspective only. Although it was recognized that gender could influence the impacts of NCDs, interviewees from the labour, legislative, judicial and private sectors said they do not apply a gender perspective beyond their institutional and regulatory limits.

Other obstacles mentioned by the interviewees were the lack of learning about the continuity of healthcare programmes, and its impact on collaboration between NGOs and government institutions, as was stated by two people from the academic sector:

*[…] When the people responsible for certain areas leave or there is a new 6-year term, it’s starting all over again. The specific action programmes with a gender perspective on health are wiped out, just like that […] (Interview 11, Woman, academic sector)*

*[…] It’s not lack of learning, but of resources and political will. Gender perspective has diluted in the last few years, despite what it is said […] (Interview 9, Woman, academic sector)*

Despite this, interviewees from the academic, legislative, judicial and labour sectors agreed that there is a will to work collaboratively. They also pointed out that there is now a greater proportion of women in leading positions, following the development of political instruments such as treaties, agreements and laws.

For the labour and legislative sectors, as well as for NGOs, the strongest social and political pressure related to the gender agenda has come in relation to violence against women. NGOs emphasized the importance of strengthening their capacity for influencing the political agenda to direct more attention to NCDs, agreeing with stakeholders from the academic sector about their significant social and economic effects. In this regard, an informant from an NGO said:

*[…] In the government agenda, the priority regarding gender is sexual and reproductive health, and violence because it’s a life-or-death issue. The healthcare sector is gender-sensitized only in these topics […]. (Interview 7, Woman, NGO)*

Government actors identified some opportunities for collaboration between the government, NGOs, legislative power, and the private sector (see Table 5). These interviewees suggested the possibility of integrating funds and operational departments to improve the system’s response for people living with some NCDs. In financial terms, NGOs could receive more support and opening from the private sector, and the social security sector suggested inter-sectoral and collaborative work with other governmental agencies regarding gender and NCDs.

## Discussion

This study highlights the need to include NCDs in the current framework of health and gender policies in Mexico and identifies obstacles to including a gender perspective in health policies and programs aimed at reducing the burden of these diseases. The critical review of the political, health and gender agendas in Mexico confirmed that general perceptions of health conditions are limited to the medical aspects of these diseases, while gendered social and economic impacts are not widely considered. The medical, social and economic aspects are not simple to separate; for example, a high proportion of people living with NCDs in LMICs, especially women, report not taking the medication they require due to costs [44].

The gendered social impacts of NCDs in LMICs reaffirms the need to improve the response of health systems regarding access to healthcare and financial protection [29]. The testimonies of key stakeholders from the academic sector, the government and NGOs highlighted the social and economic burden of several types of cancer, along with diabetes complications and cardiovascular diseases. These statements also showed that the costs of medical care for NCDs are a consequence of differential access to healthcare services and health insurance programs. Patients and their families experience more vulnerability when medical coverage does not ensure the necessary resources to address damages, complications, multi-morbidity and side effects associated with these diseases. Only 34 countries worldwide provide paid, long-term care leave, of which 4 belong to the Americas [45].

Some high-income countries make an important effort to provide care to people living with NCDs through public services or medical insurance, demonstrating an understanding of the burden that family care represents for women [46, 47]. In this vein, our results suggest the importance of adopting a gender perspective when assessing the effects of caring for sick and elderly people at home, the creation of special schedules for treatment control and monitoring of women living with an NCD and financial protection programmes for women who are the primary income-earner of their family [48]. The latter is particularly relevant to creating a national care system in Mexico, which can cost up to 1.4% of the country’s Gross Domestic Product (GDP) [49]. This system would require a gender-sensitive, inter-institutional and inter-sectoral structure [50]. In the Mexican case, health programmes with a gender perspective focussed on women’s health, while no programmes were found specifically for men’s health, despite the fact that the disease burden is greater in men than in women [7]. The creation of a National Care System would have great benefits by improving the opportunities in the educational, labour, social and political spheres for women caregivers. Reducing the burden of unpaid work would encourage them to be able to take up or retain paid jobs, because care for their family members would be provided by the National Care System [50].

This study also highlights the shortage of documents and public policies in Mexico which aim to respond with a gender perspective to the social, economic and medical effects of NCDs and need for access to treatment. Social welfare institutions call for interventions with a gender perspective, but this is not clearly reflected in most of the programs implemented. This underlines the importance of considering the adjustments in public policies derived from budget cutbacks through a gender lens, such as the cessation of the free meals programme for children in schools, the closure of daycare centres, and the lack of support for carers [51]. Policies in other countries that have been considered favourable for gender equality include maternity and paternity leave, comprehensive nutrition and adequate stimulation as early childhood interventions, and labour flexibility policies that can reduce stress, which have favourable effects on cardiovascular diseases [52]. These contrast with policies in Mexico, which are based on women’s unpaid work and only ensure access to health services for women as long as they are mothers [53].

Governmental and social attention for NCDs should not be unarticulated [54] or limited to institutions engaged in providing medical care [55]. Since both health and healthcare are shaped by gender [8], an inter-sectoral approach is needed for the creation of multi-sectoral policies that result in better and fairer responses in health to meet the needs of a group that has been systematically marginalized [56]. Gender-sensitive policies should look to reduce gender inequalities in healthcare; however, despite regulatory frameworks and international recommendations, there are few policies with that purpose in Mexico [13].

Aligning the multisectoral effort against NCDs, from a gender perspective, with the health and development agenda at all levels and sectors of society will help address the challenges of NCDs. The WHO has recommended the development of national frameworks for the prevention and control of NCDs, which allow inter-sectoral coordination from the initial planning stages to implementation, evaluation of interventions and promulgation of public policies [25, 57].

It has been suggested that gender-sensitive health policies are inter-sectoral and should prioritize emergent topics in the agenda, such as NCDs as leading causes of death and disability for women globally [58]. Nonetheless, it is important to note that most of the described actions focus only on pregnancy, while specific actions targeting women who live with NCDs are lacking [59]. This situation is similar to the one in Mexico, where several sectors delegate NCDs’ care and their social and economic effects to healthcare institutions, where most health programmes targeted at women focus on their reproductive capacity, their identity within the family and their role as carers [60], putting their welfare at risk.

Addressing the growing demand for medical attention for NCDs requires that health reforms promoted in several countries of Latin America respond decisively to the social exclusion issues created by the segmentation and fragmentation in healthcare systems [61], thus reducing the reproduction of different forms of inequality [62]. This will require fairer social protection programmes that consider different levels of vulnerability. Some of the greatest challenges to achieving universal health coverage for NCDs from a gender perspective derive from the lack of consideration of women’s representation in the labour market and the increasing number of women who become the primary earners of their families [48]. This situation does not imply that women are only entitled to support if they are not employed and are mothers.

To attain a real commitment to reduce gender-related inequities in health, we need to collect evidence that supports the design of policies from successful experiences, focussing the analysis on the understanding of sociocultural and political contexts in each case. For that purpose, we need greater investments in research and the development of interdisciplinary methods and tools that allow us to formulate and target gender-transformative health policies which seek to address gender inequality while supporting women’s health [13].

Addressing gender inequities and improving healthcare requires a comprehensive action agenda, reflected in public policies and the commitment of stakeholders across all sectors. A multi-sectoral approach is needed to create synergistic benefits with actions in other areas, such as workplace reform, addressing gaps in data, eliminating gender bias in research, mainstreaming gender budgeting, funding civil society organizations and social movements, and strengthening accountability mechanisms in government [63].

This study was not exempt from limitations. We did not have access to informants from the labour and judicial sectors to discuss a more inter-sectoral approach. We could not cover all the people in our directory, and we missed the perspective of key stakeholders from the media. There could be a potential bias in the responses from stakeholders, who could be influenced by their political positions. Finally, it is possible that there was gender bias in the testimonies collected due to the imbalance in the informants’ gender (almost 90% were women). Addressing these limitations could allow the identification of more favourable and receptive scenarios, support the attraction of more comprehensive attention to the impact of NCDs on women’s health, and help to develop better policy instruments. Since this study is novel in the Americas, it can become a reference point for future studies to develop a regional perspective on the topic analysed.

## Conclusions

The lack of gender-sensitive public policies and the absence of gender-sensitive budget priorities show that the main obstacles to a gendered approach to NCDs in Mexico are political and economic. The burden of care and financial vulnerability are influenced by gender affecting the social management of NCDs; these elements must be considered by the State to improve gender equality and equity. There are opportunities to innovate in health policies in the labour and law enforcement areas, which requires a comprehensive review of legal aspects to prevent setbacks in meeting healthcare needs, to decrease systemic gender vulnerability. The growing number of women in positions of leadership in Mexico, who might be expected to welcome a more gender-sensitive approach in health policies, represents a window of opportunity, as does the willingness among different stakeholders to collaborate described in the results section, as this could support an intersectional, multi-sectoral approach.

## Data Availability

The data analysed during the current study are available from the corresponding author upon reasonable request.

## Appendix 1

### Complementary material 1. Search strategy

We used the following terms and keywords for the search: gender, policies, Mexico, health, equity, equality, medical services, medical insurance, diabetes, hypertension, cancer, gender in healthcare, health services, women, men, programs, chronic diseases, cardiovascular diseases and chronic respiratory diseases.

The websites chosen for searching documents or publications appear below.

**Table:**

Websites explored	URL	Justification
Mexican government	https://www.gob.mx/	• Reports to apply gender equality and transversality perspectives and public policies
Secretariat of Health	https://www.gob.mx/salud	• Reports to apply gender equality and transversality perspectives and public policies • Promotes the improvement of reproductive rights • Regarding NCDs, highlights cervical and breast cancer prevention • Promotes gender violence prevention
Secretariat of Public Education	https://www.gob.mx/sep	• Reports to apply gender equality and transversality perspectives and public policies • Promotes an education model with a social inclusion perspective
Secretariat of Labour and Social Welfare	https://www.gob.mx/stps	• Reports to apply gender equality and transversality perspectives and public policies • Reports the development of a “new work culture”, with sections on gender in labour relations
Secretariat of Welfare	https://www.gob.mx/bienestar	• Reports to apply gender equality and transversality perspectives and public policies
Chamber of Deputies	http://www3.diputados.gob.mx/	• Reports to apply gender equality and transversality perspectives and public policies • Highlights a legislative model with a representativity and social inclusion approach
Mexican Senate	https://www.senado.gob.mx	• Reports to apply gender equality and transversality perspectives and public policies • Highlights a legislative model with a representativity and social inclusion approach
Mexican Institute of Social Security	http://www.imss.gob.mx/	• Reports to apply gender equality and transversality perspectives and public policies • Highlights the coverage of NCDs and their complications, established in the labour agreement • Describes preventive interventions for cancer, diabetes, and cardiovascular diseases
Institute for Social Security and Services for State Workers	https://www.gob.mx/issste	• Reports to apply gender equality and transversality perspectives and public policies • Highlights the coverage of NCDs and their complications, established in the labour agreement • Describes preventive interventions for cancer, diabetes and cardiovascular diseases
National Institute for Women	https://www.gob.mx/inmujeres	• Reports to apply gender equality and transversality perspectives and public policies • Promotes the improvement of reproductive rights • Regarding NCDs, highlights cervical and breast cancer prevention • Promotes gender violence prevention
National Centre of Gender Equality and Reproductive Health	https://www.gob.mx/salud/cnegsr	• Reports to apply gender equality and transversality perspectives and public policies • Promotes the improvement of reproductive rights • Regarding NCDs, highlights cervical and breast cancer prevention • Promotes gender violence prevention
UN Women	https://mexico.unwomen.org/es	• Entity of the United Nations that promotes gender equality and women empowering • Promotes to accelerate progress that will lead to improve women’s life conditions

Source: Own preparation.

To search on Google, we used the term “Mexico” along with Boolean operators “AND” and “OR” and the terms “policies”, “program”, “gender” and “health”, and the key works selected, as shown in the following example:

Mexico AND (policy OR program) AND gender AND health AND (equity OR equality OR "medical services" OR "medical insurance" OR "gender in health" OR "health services") AND (women OR men) AND (diabetes OR hypertension OR cancer OR "chronic diseases" OR “cardiovascular diseases" OR "chronic respiratory diseases").

Keywords formed by two or more words we put in quotation marks so that the search engines of the websites could identify documents, papers or publications with those specific terms, without segmenting them, to avoid the results were not viable.

## Appendix 2

### Complementary material 2. Interview guide

**Objective:** To characterize the scope of the contents of public policies related to gender to map their implications for health and non-communicable diseases (NCDs), as well as the obstacles and opportunities to attain greater gender equality in Mexico.

**Field strategy:** Operating in parallel to the scheduled review of government documentation, we made a directory with the contact information of officers, lawmakers, activists and NGOs related to several levels of gender policies (incidence, formulation, implementation and assessment). Once the directory was done, we proceeded to send the candidates for the interviews the respective official invitations by email. This invitation was made in INSP headed paper, directed to the person responsible, and signed by the main researcher; in this document, the candidate was informed of the project and attached to the interview guide and the informed consent letter. The candidates were given the choice to interview in person, following safety protocols due to the COVID-19 pandemic, or through the digital platform of their choice (Zoom, WhatsApp or Google Meet etc.).

**Application of the interview guide:** To conduct the interviews, the field personnel must be punctual and behave properly to create a trustful environment in order to talk following the informants’ autonomy and availability protocols. Also, they must ask for authorization to take notes and record the conversation. For that purpose, they must explain in detail how the data will be processed and his or her personal information will be protected. In all personal interactions, they must take measures to reduce the risk of contagion of the SARS-CoV-2 virus, prioritizing open places or with excellent ventilation, a minimum physical distance, permanent and proper use of high-quality facemasks, plus the measures requested. The informants must keep in mind that their participation is voluntary and that supporting the project does not represent any benefit to them. Likewise, it must be clear that the information will be handled with confidentiality by a very reduced number of people, looking to eliminate beforehand all the data that make the informants identifiable.

Each person was asked the following questions:

## I. Objectives of gender policies in health and NCDs

What are the most remarkable contents regarding gender of the programme/policy at your charge? (Inquire into specific aspects related to their contents, requesting some examples, if possible).

What are the mechanisms and strategies of the programme/policy at your charge to promote gender equality?

How directly or indirectly does the program/policy at your charge influence healthcare? Do you particularly take into account aspects related to NCDs (absenteeism, special schedules and scheduled medical appointments)?

## II. Inter-institutional coordination

Do you work in coordination with other institutions and organizations for the implementation, monitoring or assessment of the programmes/policies at your charge?

What regulatory changes do you consider relevant to make progress in the fulfilment of the objectives of the programmes/policies at your charge?

Which have been the most remarkable administrative innovations that you have implemented for the fulfilment of the programmes/policies at your charge?

What administrative entities were created to strengthen this coordination?

## III. Resources

To what extent do you consider that the regulations and mechanisms for the allocation of financial resources for gender equality policies have been adequate?

To what extent do you consider that the regulations and mechanisms for the allocation of financial resources can improve the performance of gender equality policies?

How do you assess the ability and capacity of health services to create financial resources to handle and manage NCDs?

Have you developed any state strategy regarding human resources to improve the management of financial resources for the population with no social security?

## IV. Political dimensions of federalism

How has the political party system influenced the way gender and health policies and programmes are handled?

Which federal and state stakeholders and institutions have been the most influential in this process?

What have been the main obstacles to achieving the objectives of the gender and health programmes and policies in Mexico?

Which have been the main opportunities to achieve the objectives of the gender and health programmes and policies in Mexico?

Do you want to add any other comments?

## Sociodemographic features

Age, academic level, gender, position and brief professional trajectory.
